# Risk factors for acute respiratory tract infections in general practitioner patients in The Netherlands: a case-control study

**DOI:** 10.1186/1471-2334-7-35

**Published:** 2007-04-27

**Authors:** Arianne B van Gageldonk-Lafeber, Marianne AB van der Sande, Marie-Louise A Heijnen, Marcel F Peeters, Aad IM Bartelds, Berry Wilbrink

**Affiliations:** 1 National Institute of Public Health and the Environment, Centre for Infectious Disease Control, Epidemiology and surveillance, Bilthoven, The Netherlands; 2 Netherlands Association for Community Health Services, Utrecht, The Netherlands; 3 Regional Laboratory of Public Health, Tilburg, The Netherlands; 4 Netherlands Institute for Health Services Research, Utrecht, The Netherlands; 5 National Institute of Public Health and the Environment, Centre for Infectious Disease Control, Diagnostic Laboratory for Infectious Diseases and Perinatal Screening, Bilthoven, The Netherlands

## Abstract

**Background:**

Acute respiratory tract infections (ARTI) are an important public health problem. Improved identification of risk factors might enable targeted intervention. Therefore we carried out a case-control study with the aim of identifying environmental risk factors for ARTI consultations in the Dutch general population.

**Methods:**

A subset of patients visiting their GP in the period of 2000–2003 with an ARTI (cases) and age-matched controls (visiting for other complaints) were included in a case-control study. They were asked to complete a questionnaire about potential risk factors. Conditional logistic regression was used to calculate odds ratio's (OR) and 95% confidence intervals (CI) to estimate the independent effect of potential risk factors.

**Results:**

A total of 493 matched pairs of case and control subjects were enrolled. Exposure to persons with respiratory complaints, both inside and outside the household, was found to be an independent risk factor for visiting a GP with an ARTI (respectively OR_adj _= 1.9 and OR_adj _= 3.7). Participants exposed to dampness or mould at home (OR_adj_=0.5) were significantly less likely to visit their GP. In accordance with the general risk of consultations for ARTI, participants with a laboratory-confirmed ARTI who were exposed to persons with respiratory complaints outside the household were also significantly more likely to visit their GP (OR_adj_=2.5).

**Conclusion:**

This study confirmed that heterogeneity in the general population as well as in pathogens causing ARTI makes it complicated to detect associations between potential risk factors and respiratory infections. Whereas it may be difficult to intervene on the risk posed by exposure to persons with respiratory complaints, transmission of ARTI in the general population might be reduced by improved hygienic conditions.

## Background

Acute respiratory tract infections (ARTI) are an important public health problem. Worldwide they are responsible for considerable morbidity and mortality, and lead to an increase in absence from work and school and an increased number of consultations with clinicians [1-6]. Based on a population of 16 million individuals, it is estimated that, in the Netherlands, almost 900,000 persons annually visit their GP for an ARTI [4].

Although antibiotics are effective against bacteria, many ARTI are caused by a multitude of other micro-organisms, mainly viruses [4,5,7-10]. The development of an effective universal treatment for ARTI is hampered by this great number of different pathogens causing ARTI as well as a diagnostic deficit of around 30% [7]. Symptomatic management therefore remains the only therapeutic option. The development of preventive initiatives to reduce morbidity and mortality, like vaccines or antiviral agents, is also hindered by the mixed pathogenesis [1,11].

Another approach towards prevention is to intervene in risk factors contributing to respiratory infections. Identification of these risk factors may be useful for efforts to interrupt transmission. A multitude of studies have identified various risk factors for ARTI. Studies in developing countries have identified risk factors to be among others crowding, nutritional factors, and parental smoking [12-15]. Because of major differences in living conditions and environmental circumstances these study outcomes can not directly be extrapolated to industrialised countries. Studies in industrialised countries can roughly be divided into studies addressing children, and studies addressing specific respiratory diseases. Known risk factors for children are for instance young age [16], environmental tobacco smoke [17,18], home-dampness [19,20], and attending day-care centres [19,21,22]. Risk factors noted in relation with specific respiratory diseases, like asthma, COPD and tuberculosis, are active smoking, low socio-economic status, occupational exposure and exposure to air pollution [23-28].

However, little is known about the role of risk factors for ARTI in the general population in industrialised countries. To contribute to the present knowledge of ARTIs we have carried out a case-control study with the aim of identifying environmental risk factors in the Dutch general population.

## Methods

This study was performed in collaboration with the network of general practices in the Continuous Morbidity Registration of the Netherlands Institute of Primary Health Care (NIVEL). This network constitutes a representative group of about 67 general practitioners (GPs) in 45 practices. Their patient population accounts for approximately 1% of the Dutch population and is representative with regard to age, sex, regional distribution, and degree of urbanization.

From October 2000 through October 2003 all patients who presented with ARTI were classified by week of consultation and age to obtain stratified incidence rates. In addition in this period, almost half of the practices (22 in 2000–2001; 19 in 2001–2002; and 18 in 2002–2003), consented to participate in a case-control study [4]. Patients were classified by the GP's. Inclusion criteria for cases were: consulting GP for acute respiratory complaints, and being diagnosed as influenza-like illnesses (ILI) or another ARTI, and consulting GP for the first time in that episode. Patients were not eligible for inclusion when they reported use of antibiotics or anti-viral medication in the last two weeks. For each case patient a control patient, matched by age group (0–4, 5–14, 15–24, 25–44, 45–64 and ≥ 65 year), was recruited within 1 week of consultation. Inclusion criterion for controls was a consultation of the GP for complaints other than respiratory. Exclusion criteria were: complaints of an ARTI in the last two weeks, belonging to the same household as the case, and the use of antibiotics or anti-viral medication in the last two weeks. From all participants, 1 nose and 2 throat swabs were obtained during the visit. Furthermore, both cases and controls were asked to complete a detailed questionnaire about potential risk factors. These questionnaires extracted information about exposure to persons with respiratory complaints within or outside their household in the week before consulting the GP, family composition (number of children and adults, presence of children attending day-care, primary school or secondary education), working outside the home and kind of job, use of public transport, type of heating system, exposure to mechanical ventilation system, exposure to dampness or mould at home, keeping pets or cattle, smoking behaviour, and exposure to passive smoking (see Additional file 1). Parents or guardians were asked to complete questionnaires for young children. To assess the burden of the ARTI, the questionnaires of case patients also extracted information about restriction of daily activities, bed rest, and absence from school or work. Moreover, after the first and second year of study all GPs participating in the case-control study were asked to complete a questionnaire for each reported case patient. These follow-up questionnaires obtained information about additional consultations, referral to specialists and hospitalisation for the reported episode of ARTI.

### Laboratory methods

Viral culture and polymerase chain reaction (PCR) were performed at the National Institute of Public Health and the Environment. Both nose and throat swabs were tested for adeno-, corona-, entero-, human metapneumo-(hMPV), influenza-, para-influenza-, rhino-, and respiratory syncytial (RS) virus, and also for *M. pneumoniae*, *C. pneumoniae and C. psittaci *[29-32]. Bacteriological cultures were performed at the Regional Public Health Laboratory in Tilburg using the throat swabs to detect bacterial pathogens known to cause 'community acquired' respiratory infections [4].

### Statistical methods

Apart from the original matching at the selection of cases and controls, a secondary matching was done to match single cases and controls. First of all for every single case a control was sought in the same age group, the same practice and within a period of three months. If no control could be selected within the same practice, a control from a different practice was selected.

Associations with potential environmental risk factors were assessed by comparing all case patients with control patients matched by age. A sub analysis addressed only these case patients in whom at least pathogen was detected and their matched control patients. Conditional logistic regression was used to calculate odds ratio's (OR) and 95% confidence intervals (CI) to estimate the independent effect of potential risk factors. Sex was included in the multivariate regression model in order to adjust for confounding. Variables were excluded from the regression model if the crude OR was not significant (P > 0.2). An exception was made for variables with respect to smoking because of the existing data on of adverse health effects of (passive) smoking [33-35].

## Results

In total, nose and throat swabs were obtained from 645 case patients and 558 control patients. Three hundred and thirty two (332) originally matched pairs of case patients and control subjects could be included. After the secondary matching, an additional 162 pairs could be formed, thus 493 pairs were included in the matched analyses. Characteristics of case patients and control subjects are summarized in table 1.

**Table 1:** Baseline characteristics of the participants in the case-control study on acute respiratory tract infections in the general practice network, 2000–2003

Characteristics	Case patients (n = 493)	Control subjects (n = 493)
Sex (%)		
Male	46	37
Female	54	63
Age, mean (years) [P25–P75]	36 [22–49]	37 [22–52]
Received influenza vaccination for the season (%)		
yes	16	14
No	84	86

**Figure 1:**
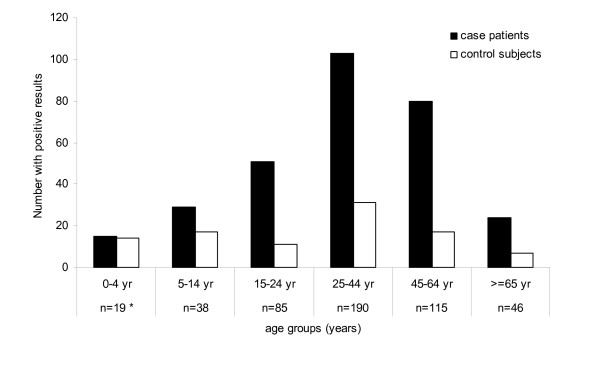
Number of nose and throat swabs in case patients and control subjects that tested positive for one or more pathogens, by age group. * number of matched case-control pairs.

Viruses were detected in 248 of the 493 case patients (50%), β-haemolytic streptococci in 41 (8%), and mixed infections in 13 case patients (3%). Influenza and rhino viruses were the most common pathogens. No pathogens were detected in 191 of the 493 case patients (39%). Moreover, pathogens were detected in 97 of the 493 control subjects (20%). The percentage of nose and throat swabs obtained from case patients with ARTI and from control patients that tested positive for one or more pathogens, according to age group, is shown in figure 1. In general, the proportion of positive nose/throat swabs decreased with increasing age group for both case patients and control subjects. These data have been described in more detail previously [4].

Perceived restrictions in daily activities were reported by 79% of the case patients with a median duration of 5 days. Both bed rest and absence from school or work were reported by approximately 65% of the cases patients (median duration respectively 2 and 5 days). These findings apply for all case patients as well as for those with a laboratory-confirmed ARTI.

A total of respectively 259 and 165 follow-up questionnaires were received after the first and second year of study. All questionnaires were included in the analyses, whether or not cases fulfilled the in-/exclusion criteria. In the first year of study 66 of the 259 case patients (25%) consulted their GP more than once for the same episode. For 47 of these 66 (71%) medication, like antibiotics, codeine, and prednisone, was prescribed at a later stage. Three patients were referred to an ENT specialist, two to a lung specialist and two to a paediatrician. Three patients were hospitalized for an ARTI, of which two with tonsillitis and one with pneumonia. In the second year of study 43 of the 166 case patients (26%) consulted their GP more than once, of which 30 (70%) were prescribed medication. Two patients were referred to an ENT specialist and three to a lung specialist. Five patients were hospitalized for an ARTI (2 tonsillitis and 3 pneumonia).

### Risk factors

#### All cases vs. matched controls

Table 2 presents the results of the univariate and multivariate risk factor analyses for all case patients consulting their GP for an ARTI compared with their matched control subjects. In the univariate risk factor analyses no significant association was observed for number of adults in the household, belonging to families with children attending day-care centres, working outside the home, use of public transport, type of heating system, keeping pets or cattle, and educational level. Therefore these potential risk factors were not included in table 2.

**Table 2:** Univariate and multivariate conditional logistic regression analyses of potential risk factors for consulting the general practitioner with an acute respiratory tract infections.

Potential risk factor	Case patients n/N (%)	Control patients n/N(%)	Crude OR* [95%CI]*	Adjusted OR** [95%CI]
Exposure to respiratory complaints within household				
No	168/283 (59)	218/283 (77)	1	1
Yes	115/283 (41)	65/283 (23)	2.32 [1.58–3.39]	**1.91 [0.96–3.78]**
Exposure to respiratory complaints outside household				
No	112/330 (34)	208/330 (63)	1	1
Maybe	87/330 (26)	47/330 (41)	3.40 [2.17–5.37]	**3.85 [1.66–8.93]**
Yes	131/330 (40)	75/330 (23)	3.50 [2.31–5.29]	**3.65 [1.70–7.84]**
Number of children in household				
0	166/340 (49)	184/340 (54)	1	1
≥1	174/340 (51)	156/340 (46)	1.37 [0.95–1.98]	1.42 [0.51–3.95]
Children in primary school				
No	137/220 (62)	151/220 (69)	1	1
Yes	83/220 (38)	69/220 (31)	1.52 [0.93–2.47]	1.12 [0.43–2.91]
Children in secondary education				
No	166/217 (76)	153/217 (71)	1	1
Yes	51/217 (24)	64/217 (29)	0.67 [0.41–1.10]	**0.32 [0.13–0.77]**
Dampness or mould at home				
No	266/313 (85)	244/313 (78)	1	1
Yes	47/313 (15)	69/313 (22)	0.63 [0.42–0.95]	**0.48 [0.23–1.00]**
Mechanical ventilation system				
No	138/333 (41)	163/333 (49)	1	1
Yes	195/333 (59)	170/333 (51)	1.36 [1.00–1.86]	1.31 [0.75–2.27]
Smoking by participant				
No	180/354 (51)	184/354 (52)	1	1
In past	85/354 (24)	78/354 (22)	1.14 [0.75–1.74]	1.25 [0.56–2.80]
Yes	89/354 (25)	92/354 (26)	1.00 [0.67–1.49]	0.91 [0.40–2.07]
Exposure to passive smoking				
No	230/355 (65)	221/355 (62)	1	1
Yes	125/355 (35)	134/355 (38)	0.89 [0.65–1.22]	0.56 [0.27–1.13]

^# ^GP = general practitioner

^## ^ARTI = acute respiratory tract infections

* OR = odds ratio; CI = confidence interval

** adjusted for all variables and sex

Exposure to persons with respiratory complaints, both inside and outside the household, was found to be an independent risk factor (respectively OR = 1.9 and OR = 3.7 after adjustment for other relevant factors). Persons who reported to be uncertain about their exposure were significantly more prone to visit their GP with complaints due to ARTI then persons without such exposure (OR_adj _= 3.9). Participants belonging to families with children in secondary education had a significantly lower risk of consulting a GP with ARTI (OR_adj _= 0.3). In addition, participants exposed to dampness or mould at home were significantly less likely to visit their GP with an ARTI (OR_adj _= 0.5).

Apart from the overall smoking behaviour we also looked at the extent of reported smoking. The participants were divided into five classes based on the daily number of smoked items (including cigarettes, cigars and/of pipes). We compared non-smokers with participants smoking <5, 5–9, 10–19, and ≥20 items daily. Participants reporting smoking less than 5 items a day had a significantly higher risk of consulting a GP (OR_adj _= 4.6 [1.2–17.9]) compared with non-smokers. In contrast, among participants smoking of 5 or more items a day, the risk of a consultation for ARTI was not significantly different from that of non-smokers (OR = 0.9 [0.3–2.6], 0.7 [0.3–1.8] and 0.7 [0.2–2.1] when the daily number of smoked items is respectively 5–9, 10–19 and ≥20).

#### Cases with a laboratory-confirmed ARTI vs. matched controls

Table 3 presents the results of the univariate and multivariate risk factor analyses for a sub analysis of case patients with a laboratory-confirmed ARTI (i.e. ≥1 pathogen detected) and their matched control patients. In the univariate risk factor analyses no significant association was observed for number of adults in the household, belonging to families with children attending day-care centres, primary or secondary school, working outside the home, use of public transport, type of heating system, keeping pets or cattle, and educational level. Therefore these potential risk factors were not included in table 3.

**Table 3:** Uni- and multivariate conditional logistic regression analyses of potential risk factors for an acute respiratory tract infections with ≥1 detected pathogen.

Potential risk factor	Case patients n/N (%)	Control patients n/N(%)	Crude OR* [95%CI]*	Adjusted OR** [95%CI]
Exposure to respiratory complaints within household				
No	100/173 (58)	133/173 (77)	1	1
Yes	73/173 (42)	40/173 (23)	2.43 [1.50–3.96]	1.51 [0.80–2.82]
Exposure to respiratory complaints outside household				
No	70/204 (34)	125/204 (61)	1	1
Maybe	56/204 (28)	28/204 (14)	3.46 [1.96–6.10]	**3.49 [1.59–7.79]**
Yes	78/204 (38)	51/204 (25)	3.15 [1.83–5.41]	**2.45 [1.19–5.04]**
Number of adults in household				
1	18/215 (8)	32/215 (15)	1	1
≥2	197/215 (92)	183/215 (85)	1.82 [1.01–3.29]	1.38 [0.51–3.72]
Number of children in household				
0	98/216 (45)	116/216 (54)	1	1
≥1	118/216 (55)	100/216 (46)	1.69 [1.04–2.75]	0.97 [0.47–1.98]
Dampness or mould at home				
No	163/194 (84)	150/194 (77)	1	1
Yes	31/194 (16)	44/194 (23)	0.67 [0.41–1.09]	**0.60 [0.31–1.18]**
Mechanical ventilation system				
No	84/208 (40)	107/208 (51)	1	1
Yes	124/208 (49)	101/208 (49)	1.59 [1.06–2.37]	1.25 [0.72–2.17]
Smoking by participant				
No	118/223 (53)	123/223 (55)	1	1
In past	51/233 (23)	46/223 (21)	1.20 [0.71–2.03]	0.81 [0.37–1.74]
Yes	54/223 (24)	54/223 (24)	1.08 [0.65–1.79]	0.79 [0.34–1.86]
Exposure to passive smoking				
No	137/222 (62)	135/222 (61)	1	1
Yes	85/222 (38)	87/222 (39)	0.96 [0.66–1.41]	0.89 [0.50–1.59]

^# ^GP = general practitioner

^## ^ARTI = acute respiratory tract infections

* OR = odds ratio; CI = confidence interval

** adjusted for all variables and sex

In line with the general risks of consultations for ARTI, participants with a laboratory-confirmed ARTI who were exposed to persons with respiratory complaints outside the household were also significantly more likely to visit their GP (OR_adj _= 2.5). Also persons with a laboratory-confirmed who reported to be uncertain about their exposure were significantly more prone to visit their GP then persons without such exposure (OR_adj _= 3.5). In contrast, exposure to persons with respiratory complaints inside the household and exposure to passive smoking as well as to dampness or mould at home were not significantly associated with laboratory-confirmed ARTI, while being associated with consultations for complaints due to any ARTI. With respect to the extent of smoking we again found that participants smoking less than 5 items a day were at higher risk for ARTI with a microbiological pathogen identified (OR_adj _= 6.9 [1.1–41.8]) compared with non-smokers. For participants smoking 5 or more items a day this was not significantly different from that of non-smokers (OR = 0.7 [0.2–2.2], 0.5 [0.2–1.4] and 0.4 [0.2–1.1] when the daily number of smoked items is respectively 5–9, 10–19 and ≥20).

## Discussion

To our knowledge, this is the first case-control study investigating the role of risk factors for ARTI in the general population in an industrialised country. Our study showed that exposure to persons with respiratory complaints, both inside and outside the household, is a risk factor for consulting a GP with an ARTI. Only exposure outside the household appears to be a risk factor for a laboratory-confirmed ARTI. While it may be difficult to intervene on this risk factor, transmission of respiratory infections might be reduced by improved hygienic conditions. Considering the substantial morbidity it is worthwhile to investigate the feasibility and effect of intensified hygiene on illness transmission in the general population.

This study adds that risk factors found in specific study populations can not be extrapolated to the general population. We demonstrated that in the general population (passive) smoking, dampness or mould at home and having family members attending day-care were not associated with a higher risk for ARTI, which is in contrast with studies carried out in children or patients with specific respiratory diseases. The pathogenesis of ARTI involves a complex interplay between pathogens and the host's inflammatory response [1,11]. This complicated mechanism in combination with a diagnostic deficit of over 30% [7] might account for differences between risk factors found in specific study populations and in the general population.

In contrast with our findings in the general population, many studies in children concluded home dampness to be associated with increased respiratory symptoms [19,20,36,37]. Knowledge about the mechanisms behind the association between dampness and health effects and the effect of moulds on the immune system is still limited [19,38]. It cannot be excluded that the presence of dampness or mould at home in our study, based on self-reported questionnaire-data, is not observed in enough detail to assess the relation with ARTI in the general population.

Several studies describe an association of cigarette smoking or exposure to environmental tobacco smoke with the occurrence and severity of ARTI[17,18,39-41]. Smoking is believed to exacerbate respiratory diseases by harming respiratory defence mechanisms [33].  Nevertheless, in this study smoking or exposure to passive smoking was not a risk factor for consulting a GP with an ARTI nor for consulting with a laboratory-confirmed ARTI. It is possible that, selection bias is part of the explanation for this finding. Smokers might be less inclined to visit a GP with respiratory complaints as compared with non-smokers. Moreover, our findings are based on questionnaire-data about exposure, which are often prone to recall bias. Questionnaires are relatively cheap and easily used, but are likely to be less valid and reliable then the measurement of biomarkers in body fluids for true exposure [34]. Furthermore, awareness of the adverse health effects of passive smoking has increased substantially in the past years because of extensive information. This might have resulted in lower exposure levels, explaining the lack of association with ARTI in the general population. A recent study in school children also concluded that smoking by a care-provider was not significantly associated with respiratory infections [20].

Looking at the intensity of reported smoking, only *moderate smoking *(less than 5 items a day) was a risk factor for consulting a GP with a (laboratory-confirmed) ARTI in our study. The intensity of smoking is only a rough indication based on questionnaire-data, without taking into account the number of years smoking. Recall bias with regard to the extent of exposure may certainly play a part as well. Besides, it cannot be ruled out that the extent of smoking is related to care-seeking behaviour. Therefore, this finding must be interpreted with caution.

Several studies noted attendance to day-care centres to increase the risk of upper respiratory symptoms in young children [17,22]. It is plausible that day-care centre children would transmit respiratory infections to their family members [41,42]. Nevertheless, we find that having family members attending day-care, was not associated with a higher risk in the general population for consulting a GP with an ARTI. This might be an underestimation of the real number of patients with an ARTI, because an unknown proportion of subjects with an ARTI will not visit a GP. Selection bias could also play a role, persons with family members attending day-care might be less inclined to visit a GP with respiratory complaints.

Some limitations of our study should be mentioned. First the study was carried out in a period of moderate influenza-activity, affecting the number of consultations for ILI as well as other ARTI [43]. As a consequence of a limited sample size, risk factors could not be analyzed in relation to the separate pathogens, but only to the entity of ARTI. Secondly, questionnaire data were used to measure exposure to potential risk factors. This could be less reliable compared with observational data. Moreover, heightened attention to the cause of their complaints by patients with an ARTI may have caused recall bias. Thirdly, we cannot rule out the possibility that controls were in the incubation period for an ARTI, even though they had no respiratory complaints. We expect the number of controls developing those infections to be low, because the GP's actively asked for the presence of respiratory symptoms at the moment the nose and throat swabs were taken. Nevertheless, this might have diluted the investigated relations between potential risk factors and ARTI.

## Conclusion

This study showed that heterogeneity in the general population as well as in the pathogens causing ARTI in combination with the diagnostic deficit makes it complicated to observe associations between potential risk factors and respiratory infections. The risk factor found in this study, exposure to persons with respiratory complaints, can hardly be avoided. Yet better understanding of disease transmission might result in improved hygienic conditions, like hand washing more frequently, and so affect the transmission of ARTI in the general population. Increasing awareness of the importance of hygienic measures with regard to prevention and control of ARTI will also be useful facing a potential pandemic threat, assuming that the route of transmission of the pandemic virus is similar to that of other respiratory viruses.

## Competing interests

The author(s) declare that they have no competing interests.

## Authors' contributions

AG participated in the coordination of the study, performed the statistical analyses and drafted the manuscript. MS participated performing the statistical analyses and drafting the manuscript. M-LH participated in the design and coordination of the study. MP participated in the design and coordination of the study, and was responsible for the bacteriological assays. AB participated in the design and coordination of the study, and headed the network of general practices. BW participated in the design and coordination of the study, and was responsible for the virological assays. All authors read and approved the final manuscript.

## Pre-publication history

The pre-publication history for this paper can be accessed here:



## Supplementary Material

Additional File 1 Requirements: - Click here for file
