# Supplementary material for: Risk factors for acute respiratory tract infections in general practitioner patients in The Netherlands: a case-control study
Source: BMC Infect Dis. 2007 Apr 27;7:35. doi: 10.1186/1471-2334-7-35 (PMC1871593; doi:10.1186/1471-2334-7-35)
Supplement: Additional File 1 — Requirements: - [file 1471-2334-7-35-S1.doc]

**Annex 1** Detailed description of the questionnaire about potential risk factors contributing to respiratory infections

| Were you exposed to persons with respiratory complaints within your household in the week before consulting your GP? | yes / no / not applicable |
| --- | --- |
| Were you exposed to persons with respiratory complaints outside your household in the week before consulting your GP? | yes / no / don’t know |
| How many persons do belong to your household? | number of children  number of adults |
| Are there any children in your household attending day-care? | yes / no / not applicable |
| Are there any children in your household attending primary school? | yes / no / not applicable |
| Are there any children in your household attending secondary education? | yes / no / not applicable |
| Do you work outside home? | yes / no |
| If yes, what kind of job? | health care  education  government  industry  hotel or restaurant  agriculture  construction industry  retail trade  ICT  other, ……………………… |
| How often did you use public transport in the past three months? | hardly  less than once a week  once a week  more than once a week but not daily  almost daily |
| What kind of heating system do you have at home? | central heating  gas heater  space-heating  fire place  don’t know |
| Do you sometimes have dampness or mould at home? | yes / no / don’t know |
| Does your house have a mechanical ventilation system? | yes / no / don’t know |
| Do you keeping pets?  If yes, specify. | yes /no |
| Do you keeping cattle?  If yes, specify. | yes /no |
| Do you smoke at this moment or did you smoke in the past?    If yes, what is/was the daily number of smoked items? | yes, since ….  (specify date)  no, never smoked  smoked from .. to ..  (specify date)  number of cigarettes  number of cigars  number of pipes |
| Does anybody in your household smoke?  If yes, what is the daily number of smoked items in house? | yes / no / not applicable  number of cigarettes  number of cigars  number of pipes |
| How often do people not belonging to your household smoke inside your house? | hardly  less than once a week  once a week  more than once a week but not daily  almost daily |
| What is the highest school training you followed and finished? |  |
